# Cost analysis of office-based transnasal esophagoscopy

**DOI:** 10.1007/s00405-019-05357-0

**Published:** 2019-02-26

**Authors:** David J. Wellenstein, Jimmie Honings, Henrieke W. Schutte, Jasmijn M. Herruer, Frank J. A. van den Hoogen, Henri A. M. Marres, Robert P. Takes, Guido B. van den Broek

**Affiliations:** Department of Otorhinolaryngology and Head and Neck Surgery, Radboud University Medical Center, Philips van Leydenlaan 15, 6500 HB Nijmegen, The Netherlands

**Keywords:** Transnasal esophagoscopy, Office-based, Topical anesthesia, Head and neck oncology, Cost analysis

## Abstract

**Purpose:**

Although office-based transnasal esophagoscopy has been investigated extensively, a cost analysis is still lacking. We performed a cost analysis combined with feasibility study for two diagnostic processes: patients with globus pharyngeus and/or dysphagia, and hypopharyngeal carcinoma.

**Methods:**

Prospective cohort study.

**Results:**

Forty-one procedures were performed, of which 35 were fully completed. The procedure was well tolerated with mild complaints such as nasal or pharyngeal pain and burping. Four complications occurred: two minor epistaxis and two vasovagal reactions. In patients with globus pharyngeus and/or dysphagia, transnasal esophagoscopy resulted in a cost saving of €94.43 (*p* 0.026) per procedure, compared to our regular diagnostic process. In patients with suspicion of hypopharyngeal carcinoma, cost savings were €831.41 (*p* 0.000) per case.

**Conclusions:**

Cost analysis showed that office-based transnasal esophagoscopy can provide significant cost savings for the current standard of care. Furthermore, this procedure resulted in good patient acceptability and few complications.

## Introduction

Inspection of the gastrointestinal tract with a flexible fiberoptic transoral endoscope became available in the late 1950s [1]. Shortly thereafter, Hirschowitz reported on a flexible fiberoptic esophagoscope with two working channels, enabling suction or obtaining biopsies [2]. Since the 1980s, fiberoptic endoscopy has slowly been replaced by distal chip endoscopy. In these endoscopes, a charge-coupled device (CCD) chip is located in the tip of the endoscope and images are seen on a video screen [3]. Since then, addition of a working channel in the digital endoscope and enhancement of image quality has transformed the field of diagnostic and therapeutic endoscopy. Through this ongoing development, endoscopes with smaller diameters became available, and thus the first studies were published on transnasal esophagoscopy (TNE) in the 1990s [4, 5].

Since then, TNE has been extensively reported on, and has proved to accurately diagnose esophageal pathology [6–16]. Several studies showed better patient acceptability and less cardiopulmonary stress (i.e., rise in blood pressure and heart rate) during TNE, compared to transoral esophagoscopy [4, 15, 17–29]. Furthermore, TNE can be used for therapeutic office-based procedures under topical anesthesia, such as foreign body removal or esophageal balloon dilatation [30].

For otorhinolaryngologists and head and neck surgeons, inspection of the esophagus can be useful, in patients suffering from globus pharyngeus or dysphagia, to directly exclude mucosal esophageal pathology. Traditional diagnostics for these patients are limited to flexible pharyngolaryngoscopy, video fluoroscopy, rigid esophagoscopy under general anesthesia, and referral to a gastroenterologist in cases requiring flexible esophageal inspection. Furthermore, in the diagnostic workup of hypopharyngeal carcinoma, inspection of the proximal esophagus is usually performed under general anesthesia to determine the distal border of the tumor. With the introduction of TNE, examination of the esophagus for these indications can be performed in the outpatient clinic under topical anesthesia and additional indications are likely to be developed and established in the future [30, 31].

Although TNE has been extensively investigated in the last 2 decades, a cost analysis is still awaited [32]. There are several articles that reported on cost savings of TNE under topical anesthesia, but most were estimated cost savings [11, 18, 33]. Therefore, the goal of this study was to determine the actual cost difference for office-based TNE compared to two regular diagnostic processes. In patients with globus pharyngeus and/or dysphagia, the costs of TNE under topical anesthesia were compared with traditional flexible laryngoscopy and video fluoroscopy. In patients suspected of hypopharyngeal carcinoma, costs of TNE under topical anesthesia including taking biopsies were compared to the common practice of inspection under general anesthesia including biopsies. Furthermore, patient experiences and safety were evaluated.

## Materials and methods

### Patient inclusion

This prospective study was conducted in accordance with the guidelines established in the Declaration of Helsinki and was approved by the local medical ethical committee of our center (2015–2156). We estimated that 35 completed procedures would be sufficient to perform a cost analysis and investigate feasibility of office-based TNE. The first included patient category were adult patients with globus pharyngeus and/or dysphagia. All patients underwent a complete examination, including laryngoscopy, in our hospital or in the hospital from which they were referred. This patient category usually already underwent regular diagnostics (e.g., video fluoroscopy) and therapy (e.g., proton pump inhibitor or consultation of a speech language pathologist). Thus, they were examined to exclude a laryngopharyngeal or esophageal malignancy as the cause for their complaints. The second included patient category were patients with suspicion of hypopharyngeal carcinoma. These patients were referred from other (i.e., secondary) hospitals, if a suspicious tumor was seen during flexible laryngoscopy. Other hospitals refer patients immediately to reduce diagnostic delay. These patients were examined to identify the tumor, determine the distal extension in relation to the esophagus, and obtain biopsies under topical anesthesia the same day. Furthermore, they also underwent imaging diagnostics according to the national guidelines. There were no exclusion criteria.

### Setting

This study was conducted in our tertiary referral center. Patients indicated for one of the two categories were included from January to September 2016. All patients were consecutively included, until 35 completed procedures were performed. This resulted in 41 attempted procedures.

### Costs data extraction

Cost analysis was performed from a clinical diagnostic perspective, thus secondary costs (e.g., travel expenses, time off from work for patients undergoing the diagnostic process and family) and capital expenditure were not accounted for. For each of the 35 patients that underwent TNE, costs in Euros for all materials and procedures were obtained. All used materials, prices and sources are displayed in Table 1. Costs for the one-time purchase of a transnasal esophagoscope, transnasal laryngoscope and video processor, were depreciated in 5 years on an average of 50 procedures performed each year. The 35 patients were divided into two groups, the first group consisted of patients with globus pharyngeus and dysphagia (*n* = 20), and the second group consisted of patients with suspicion of hypopharyngeal carcinoma (*n* = 15).

**Table 1:** Costs for each material per patient category

Parameter	TNE (€)	Regular diagnostic process for globus pharyngeus and/or dysphagia (€)	Regular diagnostic process for hypopharyngeal carcinoma (€)
Lidocaine	0.18^a^		
Attachment for spraying	0.82^a^		
Xylometazoline	1.02^a^		
Gauze pledgets (10 units)	0.07^a^		
Biopsy forceps	17.00^a^		
Pathology container	1.29^a^		1.29^a^
Single wash of endoscope	24.00^a^		
Video fluoroscopy		281.26^a^	
Consulting pathologist	114.38^a^		114.38^a^
Surgery (half hour)			440.00^a^
1-day ward administration			476.00^b^
Outpatient clinic visit	91.00^b^	91.00^b^	91.00^b^
Single-use transnasal esophagoscope	98.87^c^		
Single-use video processor	94.68^d^	94.68^d^	94.68^d^
Single-use transnasal laryngoscope	85.12^e^	85.12^e^	85.12^e^

^a^Institution’s financial department

^b^Dutch Health Institute. Guideline for performance of economic evaluations in healthcare. February 29, 2016 version. https://www.zorginstituutnederland.nl/over-ons/publicaties/publicatie/2016/02/29/richtlijn-voor-het-uitvoeren-van-economische-evaluaties-in-de-gezondheidszorg (in Dutch). Accessed February 16, 2018

^c^Pentax Medical; €24,718.00/5 (years)/50 (patient’s per year)

^d^Pentax Medical; €23,669.00/5 (years)/50 (patient’s per year)

^e^Pentax Medical; €21.281.00/5 (years)/50 (patient’s per year)

For these two groups, the same number of consecutive patients that underwent the diagnostic process in the months before we started with TNE (i.e., the regular diagnostic process) were searched in our electronic health record system based on their medical record codes. For 20 patients with globus pharyngeus or dysphagia, the entire diagnostic process was evaluated and all costs were extracted. These patients underwent several outpatient clinic visits with laryngoscopy (VNL 1070STK, Pentax Medical, Uithoorn, The Netherlands), and if indicated video fluoroscopy (i.e., barium swallow examination). For the 15 patients with a suspicion of hypopharyngeal carcinoma, the diagnostic process before TNE was a consultation in the outpatient clinic with laryngoscopy, and afterwards an investigation (laryngopharyngoscopy and proximal esophagoscopy) under general anesthesia with biopsies and daycare admission (no overnight stay) to the inpatient ward. The average number of procedures per patient category is displayed in Table 2.

**Table 2:** Average number of products used and procedures performed per patient category

Parameter	TNE (G/D)	TNE (H)	Regular diagnostic process (G/D)	Regular diagnostic process (H)
Lidocaine	1	1	0	0
Attachment for spraying	1	1	0	0
Xylometazoline	1	1	0	0
Gauze pledgets (10 units)	1	1	0	0
Biopsy forceps	0.35	0.73	0	0
Pathology container	0.35	0.73	0	1
Single wash of endoscope	1	1	0	0
Video fluoroscopy	0	0	0.8	0.07
Consulting pathologist	0.35	0.35	0	1
Surgery (half hour)	0	0	0	1
1-day ward administration	0	0	0	1
Outpatient clinic visit	1	2	2.35	2.05
Transnasal esophagoscopy	1	1	0	0
Transnasal laryngoscopy	1	1	1.55	1.05

*G/D* globus pharyngeus and/or dysphagia, *H* hypopharyngeal carcinoma

### TNE procedure

Patients were examined in the outpatient clinic of our center. Elaborate patient instructions on topical anesthesia administration and the procedure were provided. The patient was seated, and topical nasal anesthesia was administered by placing 2–3 gauze pledgets soaked in 10% lidocaine and 0.1% xylometazoline in each nasal cavity. The gauze pledgets were left in place for a minimum of 10–15 min. Furthermore, laryngopharyngeal anesthesia was administered by applying around ten sprays of 10% lidocaine, which is lower than the maximum dose of lidocaine application in the larynx [34]. Also, the tip of the endoscope was lubricated with lidocaine gel. Patients were advised not to eat or drink until 1 h after the last laryngopharyngeal anesthesia administration, to avoid aspiration due to a desensitized laryngopharynx.

For TNE, a transnasal esophagoscope was used (EE-1580K, Pentax Medical, Uithoorn, The Netherlands). This endoscope has a 5.1 mm outer diameter with a 2.0 mm working channel, allowing suction or insufflation during examination. Digital images were processed using a video processor (EPK-i5000-HD, Pentax Medical, Uithoorn, The Netherlands).

After pharyngolaryngoscopy, the endoscope was directed into the pyriform sinus. The patient was asked to swallow, after which the endoscope was inserted into the esophagus and passed into the stomach. The stomach was inspected, including the caudal portion of the distal esophageal sphincter, by retroflexion of the tip of the endoscope, also known as the ‘J-maneuver’. Inspection of the mucosa of the esophagus was performed by retracting the endoscope slowly upwards, gaining circumferential sight of the esophagus by repeated insufflations of air through the working channel of the endoscope. In case of suspicious lesions, biopsies were taken with a flexible endoscopic biopsy forceps (Radial Jaw™ 4 pulmonary standard capacity with needle 1.8 mm diameter, Boston Scientific, Costa Rica).

After TNE, patients were asked to complete a questionnaire containing five questions regarding their experiences during the procedure. A visual analogue scale (VAS) was used to rate patients’ experiences concerning nasal pain during endoscope insertion, throat pain during examination, and inconvenience due to gag reflex, nausea, and burping. Each question is rated on a scale from 1 to 10, where 1 is the least unpleasant and 10 is the most unpleasant.

### Analysis

Statistical analysis was performed using IBM Statistical Package for Social Sciences Statistics 22 (IBM Corp. Released 2013. IBM SPSS Statistics for Windows, Version 22.0. Armonk, NY: IBM Corp). For cost analysis, the two groups were analyzed using independent-sample *t* test and bootstrapping.

## Results

Between January 2016 and September 2016, 41 TNE procedures under topical anesthesia were attempted. The results are summarized in Table 3. Cost analysis for the first group (i.e., patients with globus pharyngeus and/or dysphagia) revealed a significant cost difference in favor of TNE. The mean difference in costs was €94.43 (*p* 0.026) per procedure, with mean costs of €532.80 for TNE and €627.23 for the regular diagnostic process. This difference remained statistically significant after bootstrapping (*p* 0.035). For the second group (i.e., patients with suspicion of hypopharyngeal carcinoma), even more extensive differences were found. The diagnostic process with office-based TNE (mean costs €583.54) was significantly less expensive (*p* 0.000) compared to the regular diagnostic process (mean costs €1414.95), with a mean cost difference of €831.41 per procedure. Again, the difference remained statistically significant after bootstrapping (*p* 0.001).

**Table 3:** Patient characteristics

Characteristics	TNE	%
Study population	41	100
Sex (males)	28	68.3
Age (range)	66.6 (29–87)	
Indication		
Globus pharyngeus and/or dysphagia	26	63.4
Suspicion hypopharyngeal/esophageal carcinoma	15	36.6
Completed procedures	35	85.3
Discontinued procedures	6	14.7
No nasal passage endoscope	4	9.8
Complication	2	4.9
Duration (range minutes)	15.03 (6.38–35.00)	
Clinical findings	35	100
Globus pharyngeus and/or dysphagia	20	57.1
No suspicious lesions	11	31.4
Laryngeal cyst	2	5.7
Barrett’s esophagus^a^	1	2.9
Laryngeal carcinoma (primary or residual)^a,b^	4	11.4
Esophageal carcinoma^b^	4	11.4
Suspected hypopharyngeal tumor	10	28.6
Hypopharyngeal carcinoma	8	22.9
No suspicious lesions	2	5.7
Suspected esophageal tumor^c^	5	14.3
Esophageal carcinoma	1	2.9
No suspicious lesions	4	11.4 (4/35)
Laryngeal carcinoma^d^	1	2.9
Complication		
Epistaxis	2	4.9
Vasovagal reaction	2	4.9
VAS score patient tolerance (average)^e^		
Burping	2.2	
Pain in nose	1.9	
Pain in throat	1.7	
Gagging	1.5	
Nausea	0.3	

^a^One patient with globus pharyngeus and dysphagia had residual laryngeal carcinoma and Barrett’s esophagus

^b^One patient with globus pharyngeus and dysphagia had a tumor in the oral cavity, oropharynx and esophagus

^c^These were patients with suspected lesions that were seen on PET and/or CT

^d^One patient with a suspected esophageal tumor on PET showed no pathology in the esophagus, but residual laryngeal carcinoma

^e^Visual analogue scale (VAS): 0 = no complaints, 10 = unbearable complaints

Thirty-five procedures (85.3%) were completed. Mean VAS score for patient experience was 2.2 for burping, 1.9 for nasal pain during insertion, 1.7 for throat pain, 1.5 for gagging, and 0.3 for nausea. Six procedures were not completed, four (9.8%) due to failure of passage of the esophagoscope through the nose, and two (4.9%) due to vasovagal reaction of the patient. Both patients experienced light headedness and nausea, but did not lose consciousness, and recovered without sequelae. Two patients experienced epistaxis after TNE, which was resolved after placing cotton pledgets soaked in 0.1% xylometazoline in the nasal cavity. After a short observation, both patients fully recovered without sequelae. Of the 20 patients that underwent TNE for globus pharyngeus and/or dysphagia, 4 cases (20%) revealed primary or residual laryngeal carcinoma and for 4 cases (20%) primary esophageal carcinoma.

## Discussion

Although office-based TNE has been extensively investigated in the last decades and several studies mentioned the estimated cost savings for office-based TNE, a cost analysis was never performed [11, 18]. Therefore, we conducted this prospective clinical study to analyze the cost savings of TNE for two diagnostic indications, and investigate the feasibility at our tertiary referral center.

We demonstrated that office-based TNE provides significant cost savings in patients suffering from globus pharyngeus and/or dysphagia, in whom the primary goal was to exclude an esophageal tumor as the cause of their complaints. Even more significant cost reduction was found in patients with (suspicion of) hypopharyngeal carcinoma that underwent office-based TNE with biopsies. In these patients, examination under general anesthesia with biopsies can be omitted. As was expected, cost savings were even greater compared to cost savings for patients with globus pharyngeus and dysphagia.

An overall advantage of TNE is the favorable patient acceptance, as our own experience and several other studies have shown [30]. Only few minor complications occurred, with no long-lasting consequences for the patient. When reviewing our complications compared to the literature, we noticed higher rates of epistaxis and vasovagal reaction [30]. In patients with globus pharyngeus and/or dysphagia, 20% of the patients had esophageal carcinoma found during TNE. Although this rate was surprisingly high, similar rates have been reported in the literature [30]. Patients with globus pharyngeus and/or dysphagia that are referred to our tertiary hospital, are probably a selected group of patients and different, compared to the category of patients that are seen in a non-academic secondary referral clinic. Most esophageal pathologies were found in patients suffering from both globus pharyngeus and dysphagia, thus this combination might be a strong indication to perform TNE. An ongoing point of discussion is the screening for second primary tumors in the esophagus by esophagoscopy in patients with hypopharyngeal carcinoma, given the relatively higher incidence of esophageal carcinoma in these patients [30]. The short duration of a TNE, with few complications and good patient acceptance, could favor performing TNE, especially if the incidence rates of esophageal carcinoma are as high as found in our study.

Although we did not investigate this in our current study, our experience is that office-based TNE results in a faster diagnostic process [35]. In patients with globus pharyngeus and/or dysphagia, office-based TNE gives less burden for the patient. When no pathology is found, patients can be reassured immediately and secondary diagnostics (e.g., video fluoroscopy or referral to a gastroenterologist) can be avoided. Furthermore, TNE might replace video fluoroscopy, as it is a faster procedure, with significant cost savings and no radiation exposure. Due to this study, a significant decrease in video fluoroscopy has occurred in our center for patients with globus pharyngeus and/or dysphagia. If esophageal pathology is encountered during endoscopy, histology can also be obtained. All patients referred to us with suspicion of a head and neck malignancy are seen weekly in our multidisciplinary head and neck oncological center. With the introduction of TNE in the diagnostic process, patients with suspected hypopharyngeal carcinoma undergo TNE the same day to identify the distal border of the tumor, and biopsies are obtained. By arranging a fast diagnostic track, biopsies during TNE (instead of endoscopy under general anesthesia) are evaluated within 2 days by the pathologist and our diagnostic process has shortened from two and a half weeks, to 2 days.

Potential limitations of this study are the limited number of patients included and the lack of a power analysis, which could result in less reliable data. We chose the number of 35 participants in the context of evaluation of feasibility for TNE at our department. We performed a post hoc power analysis, that showed high power (1 − *β* 0.91) for the chosen sample size. Furthermore, indirect costs (i.e., secondary costs and capital expenditure) were not evaluated in this study, because our goal was to evaluate the cost savings from a medical perspective. By including these costs, such as travel time and time of absence from work of patients and their family, a more robust cost analysis could be performed.

In conclusion, office-based TNE resulted in significant cost savings, for patients with globus pharyngeus and/or dysphagia, and suspicion of hypopharyngeal carcinoma. Furthermore, this procedure resulted in good patient acceptance and had few complications.
